# Characteristics that make trophy hunting of giant pandas inconceivable

**DOI:** 10.1111/cobi.13458

**Published:** 2020-04-15

**Authors:** Robert A. Montgomery, Madeline Carr, Charlie R. Booher, Abigail M. Pointer, Brendan M. Mitchell, Natalie Smith, Keegan Calnan, Georgina M. Montgomery, Mordecai Ogada, Daniel B. Kramer

**Affiliations:** ^1^ Department of Fisheries and Wildlife Michigan State University 480 Wilson Road, 13 Natural Resources Building East Lansing MI 48824 U.S.A.; ^2^ College of Social Sciences Michigan State University 509 E. Circle Drive, Berkey Hall East Lansing MI 48824 U.S.A.; ^3^ James Madison College Michigan State University 842 Chestnut Rd Room S369L East Lansing MI 48825 U.S.A.; ^4^ Lyman Briggs College Michigan State University 919 E Shaw Ln East Lansing MI 48825 U.S.A.; ^5^ Department of History Michigan State University 506 E. Circle Dr East Lansing MI 48824 U.S.A.; ^6^ Conservation Solutions Afrika Muthaiga Estate P.O. Box 880–10400 Nanyuki Kenya

**Keywords:** *Ailuropoda melanoleuca*, China, conservación, recolección, *Ailuropoda melanoleuca*, China, collecting, conservation, 大熊猫 (*Ailuropoda melanoleuca*), 中国, 收藏, 保护

## Abstract

In November 1928, Theodore Jr. and Kermit Roosevelt led an expedition to China with the expressed purpose of being the first Westerners to kill the giant panda (Ailuropoda melanoleuca). The expedition lasted 8 months and resulted in the brothers shooting a giant panda in the mountains of Sichuan Province. Given the concurrent attention in the popular press describing this celebrated expedition, the giant panda was poised to be trophy hunted much like other large mammals around the world. Today, however, the killing of giant pandas, even for the generation of conservation revenue, is unthinkable for reasons related to the species itself and the context, in time and space, in which the species was popularized in the West. We found that the giant panda's status as a conservation symbol, exceptional charisma and gentle disposition, rarity, value as a nonconsumptive ecotourism attraction, and endemism are integral to the explanation of why the species is not trophy hunted. We compared these intrinsic and extrinsic characteristics with 20 of the most common trophy‐hunted mammals to determine whether the principles applying to giant pandas are generalizable to other species. Although certain characteristics of the 20 trophy‐hunted mammals aligned with the giant panda, many did not. Charisma, economic value, and endemism, in particular, were comparatively unique to the giant panda. Our analysis suggests that, at present, exceptional characteristics may be necessary for certain mammals to be excepted from trophy hunting. However, because discourse relating to the role of trophy hunting in supporting conservation outcomes is dynamic in both science and society, we suspect these valuations will also change in future.

## Abstract (es)

*Article impact statement*: Giant panda's symbolism, gentle nature, endemism, rarity, and value as an ecotourism target make trophy hunting the species unthinkable.

Características que Hacen que la Caza de Trofeos de Pandas sea Inconcebible

**Resumen**

En noviembre de 1928, Theodore Jr. y Kermit Roosevelt lideraron una expedición a China con el propósito explícito de ser los primeros occidentales en cazar un panda gigante (Ailuropoda melanoleuca). La expedición duró ocho meses y terminó con los hermanos disparándole a un panda gigante en las montañas de la provincia de Sichuan. Dada la atención simultánea en la prensa popular que describía esta expedición celebrada, se posicionó al panda gigante como un nuevo objetivo de la caza de trofeos como muchos otros mamíferos alrededor del mundo. Sin embargo, hoy en día, la caza de pandas gigantes, incluso para la generación de ingresos para la conservación, es impensable debido a razones relacionadas con la misma especie y el contexto de tiempo y espacio en el que se popularizó a la especie en Occidente. Descubrimos que el estado del panda gigante como símbolo de conservación, su excepcional carisma y temperamento gentil, rareza, valor como atracción ecoturística no consuntiva y su endemismo son integrales para explicar por qué la especie no se caza como trofeo. Comparamos estas características intrínsecas y extrínsecas con 20 de los mamíferos más comunes en la caza deportiva para determinar si los principios que aplican para los pandas gigantes pueden generalizarse para otras especies. Mientras que ciertas características de los 20 mamíferos se alinearon con las del panda gigante, muchas no lo hicieron. El carisma, el valor económico y el endemismo, en particular, fueron comparativamente únicos para el panda gigante. Nuestro análisis sugiere que, actualmente, las características excepcionales pueden ser necesarias para que ciertos mamíferos no sean objeto de la caza deportiva. Sin embargo, ya que el discurso relacionado con el papel de la caza deportiva en el apoyo a los resultados de conservación es dinámico tanto en la ciencia como en la sociedad, sospechamos que estas valoraciones también cambiarán en el futuro.

## 摘要

1928 年 11 月, 小西奥多·罗斯福和柯密特·罗斯福带领探险队到达中国, 他们的目的是成为第一批成功猎杀大熊猫 (*Ailuropoda melanoleuca)* 的西方人。这次探险持续了 8 个月, 最终两兄弟在四川省山区射杀了一只大熊猫。考虑到公众媒体对这次著名探险的大量报道, 大熊猫很可能会像世界上其它大型哺乳动物那样成为战利品狩猎的对象。而今天, 由于大熊猫物种本身及其在西方国家得到宣传普及的时空范围, 即便是为了获得保护收益而捕杀大熊猫也让人难以想象。我们发现, 大熊猫作为物种保护象征的地位、非凡的魅力及温和的性情、稀有性、作为非消耗性生态旅游资源的价值以及地方特有性, 是解释这个物种为什么没有受到战利品狩猎的必要因素。我们将大熊猫这些内在和外在的特征与 20 种战利品狩猎中最常见的哺乳动物的特征进行了比较, 分析了适用于大熊猫的原则是否能推广到其它物种身上。结果表明, 虽然受到战利品狩猎的 20 种哺乳动物的某些特征与大熊猫一致, 但有许多并不相同。特别是魅力、经济价值和地方特有性这几个因素, 均为大熊猫相对独特的特征。我们的分析表明, 目前, 某些哺乳动物若要免受战利品狩猎, 可能需要具备特殊的特征。不过, 由于在科学和社会方面对于战利品狩猎在保护成效中的作用的讨论仍在不断进行, 我们认为以上评估在未来也可能发生改变。 **【翻译: 胡怡思; 审校: 聂永刚】**

## Introduction

By the mid‐1920s, American and European hunters and collectors had launched scores of expeditions in pursuit of various specimens to populate museum and private collections. As a result, examples of almost all large mammal species across the globe had been cataloged, taxidermized, and prepared for presentation. However, there was still one elusive species inhabiting remote mountainous regions of western China about which little was known. First described in the West by French missionary and naturalist Père Armand David in 1869, it was unclear whether this large‐bodied animal with unique black and white markings was a bear or something else entirely. Following a series of inquiries in the communities of Sichuan Province, David acquired a pelt from a local hunter and taxonomically positioned the species in the genus *Ursus*, naming it *Ursus melanoleucus*, the black and white bear. Scientists later determined the species did not belong in the genus *Ursus*, but rather in a genus all to itself (Kumar et al. 2017; Sheng et al. 2018). This is one way in which the giant panda (*Ailuropoda melanoleuca*) has, and continues to, defied convention. The scant information trickling back to the West fueled excitement that a large bear‐like animal unknown to science existed in the mountains of China. Several failed collecting expeditions intending to bring back evidence of this animal intensified the anticipation.

**Figure 1:**
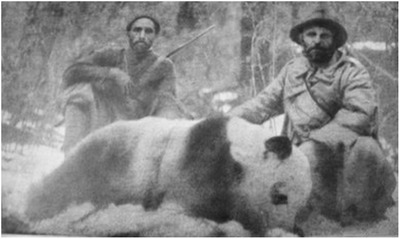
The “splendid old male” giant panda killed by the Roosevelt brothers in China in 1929 (Roosevelt & Roosevelt 1929:226). Photo courtesy of the Field Museum, Chicago (photo CSZ67964).

In 1928 Theodore Jr. and Kermit Roosevelt, sons of former U.S. President Theodore Roosevelt, left for western China as part of the Kelly–Roosevelt Field Museum Expedition. In 1929 after months of unsuccessful hunts, Theodore and Kermit simultaneously (so as to share the credit) shot a “splendid old male” giant panda (Roosevelt & Roosevelt 1929:226) (Fig. 1). Returning the pelt to the Chicago Field Museum, giant pandas were poised to become the most sought after and “challenging animal trophy on earth” (Morris & Morris 1966:46). Today, trophy hunting for giant pandas could be built on the premise that paying clients participating in a sanctioned and sustainable harvest would generate revenue necessary to conserve the species. However, trophy hunting of giant pandas as an industry has never developed.

We examined how the transition from the hunting of giant panda in the 1920s and 1930s for museum collections to live collecting for zoo exhibits drastically changed public perception of the species. We considered the unique combination of intrinsic and extrinsic characteristics that make the killing of a giant panda for sport inconceivable. These characteristics include the giant panda's conservation symbolism, purported gentle disposition, rarity, value as a nonconsumptive attraction for ecotourism, and endemism. We applied this contextual information to other species of large mammals that are trophy hunted to determine whether the principles associated with the giant panda are generalizable.

### Conservation Symbolism

Seven years after the first panda was killed by the Roosevelt brothers, and hot on the heels of 2 other high‐profile expeditions, one led by Dean Sage in 1934 and another led by Captain H. C. Brocklehurst in 1935 (Brocklehurst 1935), Ruth Harkness departed New York to become the first person to capture a live giant panda for display in the West (Harkness 1938). Ruth Harkness was an improbable person to be successful in this effort (Croke 2006), given that she was a newlywed dressmaker living in Manhattan writing about fashion trends for outlets such as the *Chicago Daily Tribune* (Harkness 1909). In 1934, Ruth's husband Bill, an amateur collector, teamed up with Larry Griswold to form the Griswold‐Harkness Asiatic Expedition. Their ambitious objective was to capture the first live giant panda and return it to the United States for display at a zoo. The expedition was doomed from the outset because Bill had an aggressive form of throat cancer that lead to his death in a Shanghai hospital in February 1936. After his death, friends of Bill convinced the grieving Ruth (hereafter Harkness) to take up the expedition in his stead.

With the support of a large team of Chinese workers, Harkness captured a giant panda cub in November 1936 deep in the remote mountains of Sichuan. By the end of the month, Harkness and the cub, Su‐Lin, greeted reporters in a Shanghai hotel room. The frenzy, later referred to as “panda‐monium,” had begun (New York Times 1938). The level of press coverage reached a fever pitch when Harkness and Su‐Lin arrived in San Francisco in December. By July of 1937, >320,000 people had visited the Brookfield Zoo in Chicago to glimpse the giant panda cub (Deuchler & Owens 2009) and *TIME Magazine* named Su‐Lin animal of the year (*TIME Magazine* 1937).

**Figure 2:**
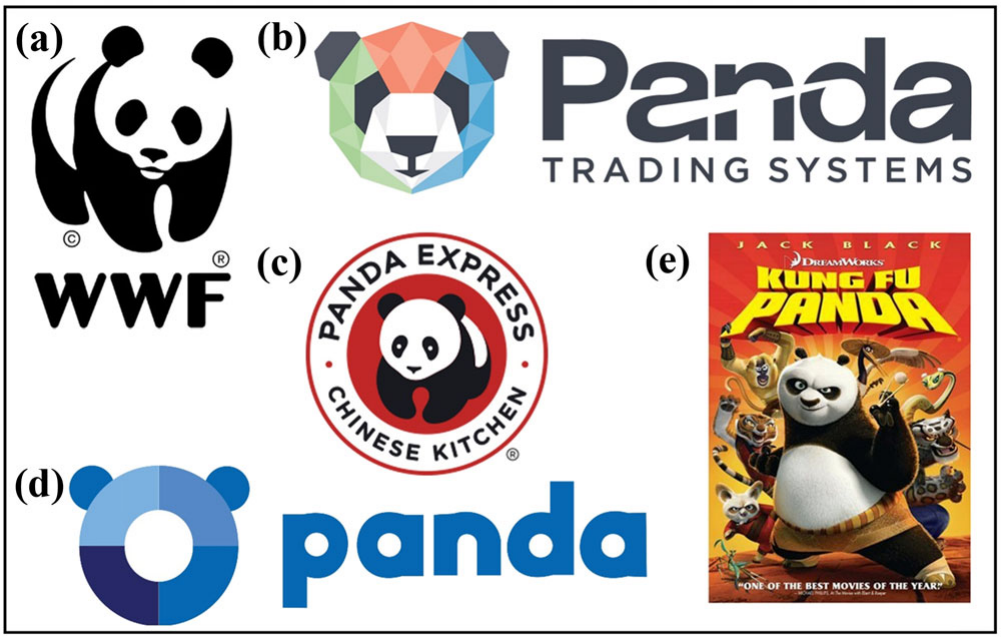
Portrayals of the giant panda (Ailuropoda melanoleuca) in marketing of the (cover in text) (a) World Wide Fund, (b) financial broker Panda Trading Systems, (c) Chinese food restaurant Panda Express, (d) information technology security company Panda Security, and (e) the DreamWorks Animated movie Kung Fu Panda.

Undoubtedly, the press, zoogoers, and the broader public were attracted to Su‐Lin's unique physical characteristics (Simberloff 1998). This attraction has much to do with the panda's body size, symmetric and circular features, and striking black eye patches (Morris & Morris 1966), all of which are intrinsically appealing to humans (i.e., *kindchenschema* [Lorenz 1943]). Cuteness is integral to marketing and fundraising campaigns (Clucas et al. 2008), and the high‐contrast image of giant pandas has been capably used to advertise films, restaurants, services, and companies (Fig. 2). In 1967 the World Wide Fund for Nature (WWF) in search of a logo snatched up the giant panda as their symbol (Greer & Doughty 1976) (Fig. 2). The WWF logo is now listed among the most iconic brands (Nicholls 2011; Bush & Oosterveer 2015). Thus, one of the reasons giant pandas are not trophy hunted today is because the species itself has become synonymous with conservation (Kontoleon & Swanson 2003).

### The Charismatic and Gentle Giant Panda

The challenge of the hunt and the ferocity of the target species are 2 primary motivations for hunters to participate in arduous, expensive, and sometimes dangerous expeditions in pursuit of animal trophies (MacKenzie 1988; Radder 2005). For example, classification as a Big Five animal (African lions [*Panthera leo*], leopards [*Panthera pardus*], African buffalos [*Syncerus caffer*], rhinoceroses [*Ceratotherium simum* and *Diceros bicornis*], and African elephants [*Loxodonta africana*]) was based on these species being the 5 most difficult to hunt and kill in Africa (Darimont et al. 2017). Thus, the challenge of hunting a species is an integral part of the trophy‐hunting experience (Hendee 1974).

In the 1920s, giant pandas certainly had a reputation of being a tough species to stalk. In describing the prospect of killing a giant panda to other would‐be hunters, the Roosevelts remarked “Owing to their scarcity and the nature of the country in which they live the prospects of getting a shot at one still hunting are very slender” (Roosevelt & Roosevelt 1929:262). Today, giant panda habitat remains high in the mountains of western China in provinces that are remote and inaccessible. Thus, giant pandas satisfy the first criterion of many trophy hunters: difficult to locate and track. However, giant pandas are far from fierce. They are one of only a handful of species in the order Carnivora that maintain an almost exclusively herbivorous diet (Nowak 2005). Thus, despite having carnassial teeth (the feature that makes a mammal a carnivore), giant pandas began to abandon meat eating 2.0–2.4 million years ago (Zhao et al. 2010; Han et al. 2019). By transitioning from a meat‐ to a plant‐based diet, giant pandas no longer had to fiercely pursue mobile and elusive prey. Although reports have occasionally highlighted aggressive behavior among captive pandas, depictions of the species as gentle animals are ubiquitous (New York Times 1939, 1943, 1966). The Roosevelt brothers stated that “The giant panda from all that we could learn is not a savage animal. After the shooting our Kashmiri shikaries remarked that he was a Sahib, a gentleman, for when hit he had remained silent, and not called out as does a bear” (Roosevelt & Roosevelt 1929:263). This distinction likely matters when it comes to trophy hunting because true bears, valued for their ferocity, are a popular target of trophy hunters (Packer et al. 2009).

The gentle reputation of the giant panda was further engrained when Harkness returned to the United States with Su‐Lin. In her hotel room in New York City, prior to Su‐Lin going on display at the Brookfield Zoo, Harkness was visited by Theodore Roosevelt Jr. and Dean Sage. Both men were changed as a result of their interaction with Su‐Lin. Theodore, for instance, suggested that putting Su‐Lin in a zoo would be comparable to placing his own child in captivity. On meeting Su‐Lin, Dean Sage commented “Do you know I shall never shoot another panda” (Harkness 1938:271). Clearly, the giant panda has a unique capacity to captivate the general public (Schuttler et al. 2019) and trophy hunters alike, due to their gentle and exceptionally charismatic nature. Thus, although the description of a panda as *giant* would no doubt be attractive to some trophy hunters, even a hard to reach and inaccessible target is not exactly sporting when the animal maintains a largely solitary, peaceful existence, and rarely exhibits direct aggression toward humans (O'Brien 1987).

Species charisma is integral to public responses to trophy hunting. Take for example the African lion, a highly sought after trophy‐hunted species (Whitman et al. 2004; Packer et al. 2009). The killing of Cecil the lion in Zimbabwe in July of 2015 spurred an incredible public backlash (Di Minin et al. 2016; Macdonald et al. 2016; Buhrmester et al. 2018). In addition to several major airlines banning the transport of lion trophies, policy makers in South Africa have proposed a ban on the captive breeding of lions for the canned hunting industry (Schroeder 2018). It is unlikely that lions would garner this level of public interest if they were not so charismatic. Among 2 separate studies on species’ charisma, lions ranked second, just behind tigers (*Panthera tigris*) (Albert et al. 2018; Courchamp et al. 2018), which is a species for which legal sport hunting is no longer permitted. Only 4 spots below lions on both of these lists is the giant panda. Thus, we infer that any proposal to open up trophy hunting for the giant panda, even in the name of conservation, would be met by unparalleled levels of public fury even when compared with the response to the killing of Cecil the lion.

### Species Rarity

**Figure 3:**
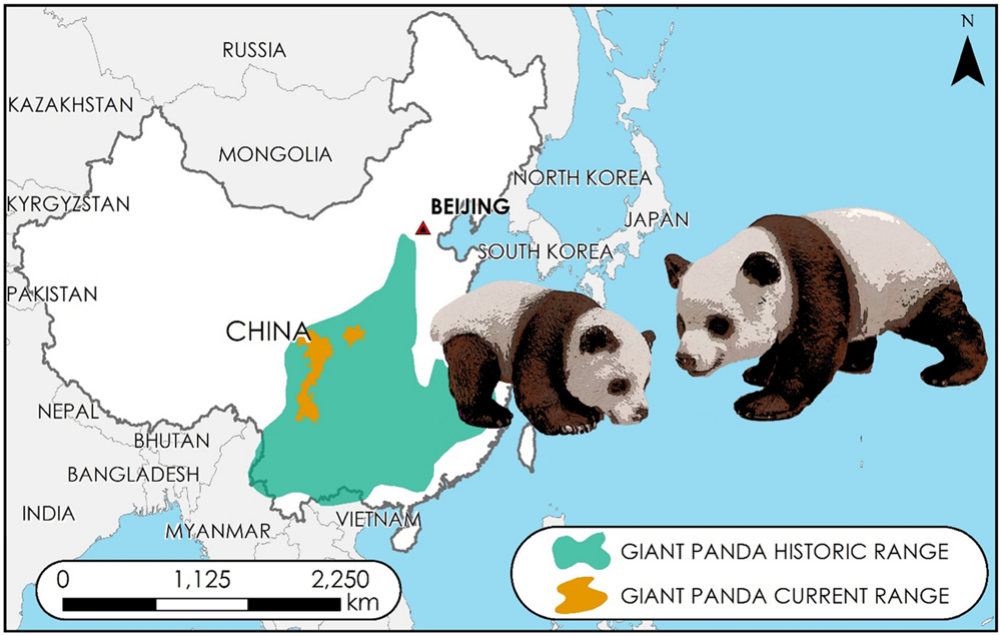
Historic and current range of the giant panda (Ailuropoda melanoleuca).

There are 500–2000 giant pandas left in the wild (Wei et al. 2018). Despite this low number, the species’ conservation status was upgraded by the International Union for the Conservation of Nature (IUCN) in 2016 from endangered to vulnerable. This revision may only be temporary, however, given that the bamboo forests on which giant pandas depend are threatened by climate change and anthropogenic fragmentation (Loucks et al. 2001; Li et al. 2015). The current species range, for instance, is subdivided among 33 different populations, which will likely have genetic consequences for the species as a whole given their isolation by distance (Kang & Li 2016) (Fig. 3). There is good reason to believe giant pandas have been comparatively rare for some time (Sheng et al. 2018). Early in their expedition in 1929, one of the hunting parties led by the Roosevelt brothers included 13 experienced Chinese hunters and their dogs. Only 2 pandas had been shot by these local hunters over the course of a dozen years (Roosevelt & Roosevelt 1929:167–168). Furthermore, although Chinese art regularly depicts various species of real or mythical wildlife (e.g., tigers, dragons, fish, and deer), the giant panda is conspicuously absent (Schaller 1994; Harper 2013), appearing only after the species became popularized in the 1950s (Songster 2018).

Species rarity, however, is not necessarily a deterrent to trophy hunting. On the contrary, very rare species, such as the black rhinoceros, which is critically endangered, are among the most expensive animals to hunt on Earth (Leader‐Williams et al. 2005; Di Minin et al. 2016). In fact, there is a strong correlation when plotting species rarity against trophy‐hunting fees (Courchamp et al. 2006). This observation is in line with conventional economic principles demonstrating that the rarity of the object tends to increase the demand for that product and, correspondingly, the cost (Ekelund & Herbert 1997; Hall et al. 2008). However, in general terms, an endangered species listing should restrict the ways in which wildlife can be used, particularly in the context of consumptive recreation (Sand 2017). Thus, the rarity of giant pandas has contributed to the fact that they are not trophy hunted for conservation.

### Economic Value of Giant Pandas

Following the frenzy associated with the arrival of Su‐Lin to the United States, Harkness launched a second expedition to procure another panda. The second cub, Mei Mei, drew 42,000 visitors on the first day of its display at the Brookfield Zoo in Chicago. The incredible popularity of the giant panda led to a veritable gold rush of expeditions launched to populate zoos with examples of this enigmatic species. Untold numbers of pandas died, either in the field, in transit, or in captivity, before making it to zoos during this period in the late 1930s (Croke 2006). Harkness experienced a moral dilemma when witnessing this gold rush. Given her confliction, Harkness led an arduous journey in July of 1938 to return the last panda she captured (named Su‐Sen) back to the wild. Shortly thereafter China banned giant panda hunting, implemented an exceptionally stringent permitting processes for export, and began to present giant pandas as an emblem of China (New York Times 1939).

The 1940s were defined by a wave of panda diplomacy that continues today (Songster 2018). In 1941 China's Madame Chiang Kai‐shek gifted 2 giant pandas to the United States in appreciation of their assistance in the Second Sino‐Japanese War (New York Times 1941). Although often given in conjunction with trade deals, the ceremonies associated with the giant‐panda gifts are steeped in pomp and circumstance. For instance, when China gave 2 giant pandas, Ling‐Ling and Hsing Hsing, to the United States in 1972 following President Nixon's historic visit to China, the pandas were moved to Washington's National Zoo “under security measures as tight as if they had been Chairman Mao” (New York Times 1972). On arrival at the zoo, the pandas were greeted by first lady Pat Nixon (Burns 2016). The giant panda proved to be an ideal diplomatic symbol for the Chinese government given the species’ attractive features, peaceful nature, and endemism (Hartig 2013). In addition to the United States, China has provided giant pandas as diplomatic gifts to Russia, Mexico, Spain, France, North Korea, and Germany (Hartig 2013). The giant pandas are technically on loan to these zoos, typically for a 10‐year period and with annual fees ranging from $500,000 to $1 million per panda (Goodman 2006). Furthermore, giant pandas born in foreign zoos increase the loan amount by ∼$400,000 per animal and each cub must be sent to China within a few years of being born (Vidal 2014).

There are an estimated 400 giant pandas in captivity, and <50 animals that have been displayed outside China (Cong et al. 2014; Shan et al. 2014). China has developed a series of impressive captive‐breeding facilities in Sichuan, Fujian, Hunan, and Shaanxi provinces. The intent of these facilities is to breed and rear giant pandas in anticipation of release into the wild. These breeding centers have become important local and international tourism destinations. Woolong, perhaps the most widely known, has over 50,000 tourists a year, a number that was 4 times higher prior to the 2008 earthquake (Liu et al. 2016). Given these economic gains, China is currently investing $1.5 billion in a new reserve roughly 3 times the size of Yellowstone National Park (Associated Press 2018). Minimum estimates of the ecosystem services associated with giant panda conservation are $2.61 billion per year in China (Wei et al. 2018).

Although panda pelts did have value in the Tang Dynasty (AD 618), there is little evidence of the giant panda ever being killed for monetary gain in China (Greer & Doughty 1976). This is particularly important given that sport hunting has occurred in China, although the practice has undergone intermittent bans (Harris & Pletscher 2002; Jiang et al. 2012; Chang et al. 2017). Furthermore, Chinese clients are a growing demographic of trophy hunters pursuing a variety of targets globally and most especially in Africa (Lindsey et al. 2007, 2012). Thus, another component of the exclusion of giant pandas as targets of trophy hunting likely also relates to the perceptions of the species among the Chinese public. One effort to quantify the cultural services of giant pandas found that >75% of Chinese respondents were willing to pay an average of $31 per year for panda conservation with the most common explanations for support being “because I love pandas” and “because pandas are a national treasure” (Ma et al. 2016). It would appear then that giant pandas are an exceptional revenue generator that are much more valuable to China alive than dead.

### Country Endemism

The control of giant pandas as a living commodity has been facilitated, in part, by the species’ endism to China. Though the historic range of the species skirted several neighboring countries (Fig. 1), giant pandas are uniquely Chinese. China was never incorporated as a colony into a foreign empire like so many other countries around the world (Zanasi 2007). Under colonial rule, imperial governments administratively controlled the governing institutions, economies, and natural resources of the countries they occupied (Headrick 1981; Porter 2012). Via occupation, colonial administrators could facilitate opportunities for citizens of their European countries to have privileged, and often exclusive, access to the hunting of wildlife in the colonies (MacKenzie 1988; Ross 2017). Many of these polices were retained, in some form or another, in the neocolonial period because institutions of power were maintained by individuals with Western training and, at least in some cases, Western loyalties (Steinhart 2006; Porter 2012). Consequently, trophy hunting, featuring predominantly Western clientele, continues to be a tremendously lucrative revenue generator. These contexts lie in stark contrast to China, where colonial governments never gained a foothold to dictate land tenure structures, exportation policies, or trophy‐hunting concessions. Thus, China has been able to exert and maintain control over the ways in which giant pandas are conserved. That control was personified in 2019 when the return of 2 giant pandas from San Diego Zoo to China coincided with bitter United States–China trade negotiations (Cao 2019).

## Implications for Conservation

Not one of the intrinsic and extrinsic characteristics of the giant panda that we considered by itself fully explains why the species is not trophy hunted. Rather, we conclude that trophy hunting for giant pandas has not developed because of a combination of factors including the species iconic status, charisma, rarity, valuation, and endemism. This conclusion calls into question whether comparable characteristics exist among other species that are trophy hunted today. We developed a list of 20 trophy‐hunted species to compare with the giant panda. We started with the Big 5 (elephants, rhinos, lions, leopards, and buffalo). We considered additional large mammalian carnivores and herbivores that are popular targets of trophy hunters (Coltman et al. 2003; Packer et al. 2009; Di Minin et al. 2016). We queried the wildlife databases maintained by the Convention on International Trade in Endangered Species (CITES) of Wild Fauna and Flora. Using records from 1975 to 2018, we searched for trophy‐hunted species listed in Appendix I of the CITES databases. Like giant pandas, species in Appendix I require export and import permits for international trade. Our final list of 20 trophy‐hunted species included 9 carnivores and 11 herbivores (Table 1).

**Table 1:** A comparison of the intrinsic and extrinsic characteristics that define the giant panda's (*Ailuropoda melanoleuca*) situation in relation to 20 of the most common trophy‐hunted mammals.*a*

Common name	Scientific name	Conservation symbol (reference code)*^a^*	Charisma*^b^*	IUCN population estimate (reference code)	IUCN conservation status	Country endemism	Approximate cost to hunt (reference code)	Top trophy hunting country	Colonial occupier of that country
Giant panda	*Ailuropoda melanoleuca*	yes (1)	6	500–1,000 (2)	vulnerable	yes	not applicable	not applicable	None
Leopard	*Panthera pardus*	yes (3)	5	data deficient (4)	vulnerable	no	∼$8,500–$24,000 (5)	Zimbabwe	United Kingdom
African elephant	*Loxodonta africana*	yes (6)	3	data deficient (7)	critically endangered	no	≥$21,000 (8)	Zimbabwe	United Kingdom
Cheetah	*Acinonyx jubatus*	yes (9)	7	6,674 (10)	vulnerable	no	≥$13,000 (11)	Namibia	Germany
White rhinoceros	*Ceratotherium simum*	yes (12)	17	20,170 (13)	near threatened	no	∼$55,000–$125,000 (14)	South Africa and Namibia	United Kingdom and Germany
Markhor	*Capra falconeri*	no (15)	>20	9,700 (16)	near threatened	no	Up to $110,000 (17)	Pakistan	United Kingdom (India)
African lion	*Panthera leo*	yes (18)	2	23,000–39,000 (19)	vulnerable	no	∼$13,500–$49,000 (11)	Tanzania	United Kingdom and Germany
Black rhinoceros	*Diceros bicornis*	yes (12)	17	4,880 (20)	critically endangered	no	up to $350,000 (21)	South Africa and Namibia	United Kingdom and Germany
Caracal	*Caracal caracal*	yes (22)	>20	data deficient (23)	least concern	no	∼$1,500 (24)	South Africa and Namibia	United Kingdom and Germany
Wild Cat	*Felis silvestris*	yes (25)	>20	data deficient (26)	least concern	no	∼$780–$4,500 (27)	Namibia	Germany
Giraffe	*Giraffa camelopardalis*	yes (28)	4	68,293 (29)	vulnerable	no	∼$3,450 (8)	Namibia	Germany
Black bear	*Ursus americanus*	yes (30)	18	850,000–950,000 (31)	least concern	no	$1,500–$5,000 (32)	United States	United Kingdom
Brown bear	*Ursus arctos*	yes (33)	18	110,000 (34)	least concern	no	$2,000–$10,000 (35)	United States (Alaska)	Russia
Impala	*Aepyceros melampus*	no (36)	>20	2,000,000 (37)	least concern	no	$250–$1,000 (8,38)	South Africa and Namibia	United Kingdom and Germany
Polar bear	*Ursus maritimus*	yes (39)	8	data deficient (40)	vulnerable	no	$24,500–25,000 (41)	Canada	United Kingdom
African buffalo	*Syncerus caffer*	no (42)	>20	398,000–401,000 (43)	near threatened	no	$1,500–$30,000 (8, 44)	Tanzania	United Kingdom and Germany
Bighorn sheep	*Ovis canadensis*	yes (45)	>20	data deficient (46)	least concern	no	$5,000–$30,000 (47)	United States	United Kingdom
Argali (Marco Polo) sheep	*Ovis ammon*	yes (48)	>20	data deficient (49)	near threatened	no	$20,000–$30,000 (50)	Kyrgyzstan	Russia (USSR)
Wildebeest	*Connochaetes* spp.	no (51)	>20	data deficient (52)	least concern	no	$1,000–$1,200 (53)	South Africa and Namibia	United Kingdom and Germany
Cougar	*Puma concolor*	yes (54)	5	data deficient (55)	least concern	no	$3,500–$8,000 (56)	United States	United Kingdom
Greater kudu	*Tragelaphus strepsiceros*	no (57)	>20	300,000–350,000 (58)	least concern	no	∼$2,000 (59)	South Africa and Namibia	United Kingdom and Germany

a The 1.

b Albert et al. (2018) and Courchamp et al. (2018).

We compared the intrinsic and extrinsic characteristics of the giant panda with each of the 20 species (Table 1). We considered each species’ conservation symbolism, charisma, rarity, value as a trophy target, and endemism. We based conservation symbolism on whether the species was part of the logo of a conservation organization. We used established rankings developed by Albert et al. (2018) and Courchamp et al. (2018) to assess charisma. We used the IUCN databases to quantify rarity, conservation status, and endemism. Finally, we calculated the approximate costs to hunt each species by evaluating trophy‐hunter databases and peer‐reviewed publications (Table 1).

Like giant pandas, the majority (*n* = 15 of 20) of these species are featured in the logos of various conservation organizations, and over half (*n* = 11 of 20) are among the 20 most charismatic species on Earth (Table 1). Most (*n* = 14 of 20) have IUCN conservation status levels comparable or lower than giant pandas (i.e., vulnerable or least concern) (Table 1). Although these 20 species have certain characteristics in common with giant pandas, other factors are rather different. None of the 20 species have a range that predominantly overlaps a single country (Table 1). Furthermore, the countries associated with the highest levels of hunting for all 20 species were, at one time, under colonial rule (Table 1). Although all 20 species generate considerable revenue from trophy hunting, the top trophy‐hunting country for the majority (*n* = 13 of 20) of these species is in Africa (Table 1). Trophy hunting is big business in Africa, with an estimated $200 million dollar annual valuation and close to 20,000 clients per year (Lindsey et al. 2007). A compelling argument could be made that had giant pandas evolved on the European, North American, or African continent, the species might be trophy hunted today.

An innate charisma, perception of a gentle disposition, and endearing physical features have made giant pandas one of the most recognizable animals on the planet (Fig. 2). They are a flagship species that has become a symbol of conservation, a symbol capable of catalyzing fundraising efforts like no other animal. Consequently, giant pandas are not dependent on any form of consumptive recreation to support their conservation. Much like the species itself, the composite characteristics that define its situation are very rare. Consequently, as we have shown, these characteristics are largely not generalizable to other species in different contexts. We conclude then that, at present, exceptional characteristics are required for large mammals to be excluded from trophy hunting. However, public tolerance of trophy hunting is changing swiftly around the world (Vucetich et al. 2019). Thus, it is probable that society will shortly deem trophy hunting inappropriate for even those species that are less charismatic, iconic, or gentle than the giant panda.
